# Allogeneic Hematopoietic Stem Cell Transplantation for Congenital Immune Dysregulatory Disorders

**DOI:** 10.3389/fped.2019.00461

**Published:** 2019-11-13

**Authors:** Shahrzad Bakhtiar, Julia Fekadu, Markus G. Seidel, Eleonora Gambineri

**Affiliations:** ^1^Division for Pediatric Stem Cell Transplantation and Immunology, University Hospital Frankfurt, Frankfurt, Germany; ^2^Research Unit for Pediatric Hematology and Immunology, Division of Pediatric Hematology-Oncology, Department of Pediatrics and Adolescent Medicine, Medical University Graz, Graz, Austria; ^3^NEUROFARBA Department, University of Florence, University of Florence, Florence, Italy; ^4^Haematology-Oncology Department, Anna Meyer Children's Hospital, Florence, Italy

**Keywords:** primary immunodeficiency, immune dysregulation, alloHSCT, IPEX, LRBA, CTLA-4, IL10R, IUIS classification 2017

## Abstract

Primary immunodeficiency disorders that predominantly affect immune regulation and mechanisms of self-tolerance have come into the limelight, because at least for a subgroup of monogenetic disorders, a targeted therapy has become available. Nevertheless, their management often involves the treatment of severely compromising, refractory, multi-organ autoimmunity, leading to further increased susceptibility to infections and complications of long-term immune suppressive treatment, including the risk of malignancy. While evidence for allogeneic hematopoietic stem cell transplantation (alloHSCT) as a curative treatment option for severely affected patients by this disease category accumulates, clear indications, and guidelines for alloHSCT are lacking. Predictive and stratification-relevant tools such as disease activity scores are largely missing and often there is not a consistent genotype-phenotype correlation within the same family to facilitate the decision whether to transplant or not. In this review, we provide a literature-based update on indications and outcomes of alloHSCT for congenital immune dysregulative inborn errors of immunity according to the IUIS classification 2017.

## Introduction

The 2017 International Union of Immunological Societies Report (IUIS) (1) on Inborn Errors of Immunity classified diseases of immune dysregulation in to seven groups: familial hemophagocytic histiocytotis (FLH syndromes), FLH syndromes with hypopigmentation, regulatory T cell (Treg) defects, autoimmunity with or without lymphoproliferation, autoimmune lymphoproliferative syndromes (ALPS), immune dysregulation with colitis, and susceptibility to EBV and lymphoproliferative conditions. Herein, we provide an update on evidence, indication and modalities of alloHSCT for Treg defects (IPEX, CD25-, CTLA-4-, LRBA-, BACH2-deficiency, and STAT3 GOF); autoimmunity disorders (APECED, ITCH-, ZAP70-, TPP2-, JAK1 GOF, and Prolidase deficiency) and immune dysregulation with colitis (IL-10-, IL10Ra-, IL10Rb, and NFAT 5 deficiency) based on data from available literature via PubMed search. FLH syndromes, ALPS and primary immune deficiency syndromes (PID) with EBV susceptibility were excluded as they are discussed elsewhere in this special edition (see chapter: “Hematopoietic Stem Cell Transplantation for Primary Hemophagocytic Lymphohistiocytosis”).

## Materials and Methods

The IUIS Phenotypic Classification for Primary Immunodeficiencies (2017) (2) was used as the basis for this review. Data were collected via English-language Pubmed literature search using the name of each disorder in combination with the terms “alloHSCT” and “transplantation.” We searched for case reports and case series on each disorder, focusing on evidence for alloHSCT as a treatment for the disease. For STAT3 GOF and LRBA deficiency unpublished data were added referring to personal communications with leading authors of each ongoing study.

## Results

### Treg Defects (IPEX, CD25-, CTLA-4-, LRBA-, BACH2-Deficiency, and STAT3 GOF)

This group of diseases is dominated by conditions defined by quantitative or qualitative impairment of the Treg compartment, predisposing to severe multi-organ autoimmunity with or without susceptibility to infections (Table 1). An increased risk of malignant transformation has been reported for some of these diseases (3, 4) and should be taken into consideration when a patient is under assessment for transplant indication. Some of the affected patients might present with milder symptoms and remain stable under supportive care, nevertheless a substantial porportion of patients develop severe complications of the disease and immunosuppressive treatment over time which brings them in an unfavorable starting position to undergo alloHSCT. Therefore, early/preemptive alloHSCT should be considered at pediatric transplant centers with expertise in PID transplantation.

**Table 1:** Treg deficiencies.

**Disease (*gene*)**	**OMIM/Inheritance**	**Clinical features**	**Targeted treatment**	**Increased risk of malignancy**	**Evidence of successful HSCT (reported cases *n* < 10, 10–100, >100)**
IPEX (*FOXP3*)	300292 XL	Treg dysfunction	Sirolimus	Not reported	10–100
		Inflammatory bowel disease			
		Type 1 diabetes			
		Eczema with elevated IgE			
		Failure to thrive			
		Nephropathy			
		Hemolytic anemia			
		Thrombocytopenia, neutropenia, autoimmune thyroiditis			
		Hepatitis			
		Arthritis,			
		Alopecia			
		Neurological findings			
CD25 deficiency *(CD25*)	147730 AR	Inflammatory bowel disease	No	Not reported	Not reported
		AI cytopenias			
		Autoimmune thyroiditis			
		Growth retardation			
CTLA4 deficiency (*CTLA4*)	123890 AD	Lymphoproliferation	Abatacept	EBV+ lymphoma, gastric cancer	10–100
		Organ infiltration			
		Inflammatory bowel disease			
		Autoimmune cytopenia			
		Enteropathy			
		Hypogammaglobulinemia			
		Respiratory infections			
		Autoimmune thyroiditis			
		Arthritis			
LRBA (*LRBA*)	606453 AR	Inflammatory bowel disease	Abatacept	Gastric lymphoma	10–100
		Malabsorption			
		Failure to thrive			
		Hypogammaglobulinemia			
		Autoimmune cytopenia			
		Endocrine manifestations			
BACH2 deficiency (*BACH2*)	605394 AD	Immunoglobulin deficiency	No	Not reported	Not reported
		Intestinal inflammation			
		Autoimmunity			
STAT3 GOF (*STAT3*)	102582 AD	Early-onset lymphoproliferation	Tocilizumab	LGL leukemia	10–100
		Lymphadenopathy hepatosplenomegaly	JAK inhibitors		
		Multiorgan autoimmunity,			
		AI-cytopenias, -hepatitis, inflammatory lung disease enteropathy			
		Hypothyroidism,			
		Diabetes mellitus			
		Growth retardation (variable)			

#### IPEX

Immune dysregulation, polyendocrinopathy, enteropathy, X-linked (IPEX) syndrome is caused by mutations in *FOXP3* (Forkhead-Box-Protein P3), a transcription factor that is expressed in a subset of thymic CD4^+^ T-lymphocytes which are required to maintain tolerance, and prevent autoimmune diseases (5, 6). The clinical manifestations and the response to different therapy strategies are heterogenous, therefore treatment of IPEX patients remains challenging without standardized therapeutic approaches. Gambineri et al. performed an analysis of molecular, immunologic, and clinical features in a cohort of 173 patients presenting with IPEX-like symptoms, and based on *FOXP3* gene sequencing, grouped them into IPEX kindreds (identifiable *FOXP3* mutation) and IPEX-like kindreds (without *FOXP3* mutation). In this study 44 *FOXP3* variants including nine new variants were identified in 88 IPEX patients. Among the 85 IPEX-like patients 19 disease-associated variants were found. Even though it was not possible to differentiate major clinical features among the two groups of patients, authors proposed a simple flow chart to evaluate these patients and to find the most likely molecular diagnosis (7).

Immunosuppressive therapy, the first line therapy of IPEX patients, has been modified in the last years and new drugs have been introduced, however, alloHSCT remains the only curative therapy option. Barzaghi et al. published an international multicenter retrospective study with a long-term follow-up of IPEX patients after different therapeutic strategies. This study included 96 genetically proven IPEX patients from whom 58 patients underwent alloHSCT (8). Patients undergoing alloHSCT received both myeloablative (MAC) and reduced intensity conditioning (RIC) regimens. The majority of the patients received RIC (*n* = 37) including fludarabine plus non-myeloablative doses of busulfan, treosulfan, or melphalan or minimal intensity including fludarabine plus low dose radiation or cyclophosphamide. MAC included busulfan (AUC 80–90 mg or >14 mg/kg), cyclophosphamide, plus fludarabine, or treosulfan. Serotherapy including Alemtuzumab or ATG was used in 49 of the 58 patients. Fifteen patients died (26%), and the estimated overall survival at 15 years was 73.2%. The majority of deaths occurred early (by day +365), mainly due to infectious complications. The type of conditioning, choice of donor, and age at transplantation did not affect survival, whereas the clinical status of the patients at the time of transplant, which was graded using an organ involvement index, did have an impact on overall survival. Acute graft vs. host disease (GVHD) was reported in 19 patients (grade III or IV in 9) while chronic GVHD was observed in six of 52 patient who survived beyond 100 days. Mixed chimerism was observed in 18 patients, and in 50% (9 patients) of them it was associated with complete disease control. The T regulatory cells were 100% of donor origin in three of these nine patients. Five of the 18 patients with mixed chimerism have died, and four were reported to be alive with autoimmune manifestations. Overall, a similar remission rate was observed among patients with full (54%) or mixed (50%) chimerism. Graft failure was observed in four patients (8). Prior to transplant or in untransplanted patients, immunosuppressive therapy included combinations of systemic steroids with cyclosporine A, tacrolimus, rapamycin, azathioprine, methotrexate, mycophenolate-mofetile, mesalazine, sulfasalazine, 6-mercaptopurine, anti-TNF-α, anti-CD20, and abatacept. Ten patients had completely controlled autoimmunity after immunosuppressive therapy whereas 24 patients still had ongoing autoimmune symptoms like enteropathy, or even developed new autoimmune manifestations. Although the overall survival was 86% at 15 years, the disease-free survival was 37 for untransplanted patients vs. 56% in transplanted patients. Moreover, the persistence or the onset of new autoimmune manifestations was high in patients under immunosuppressive treatment suggesting that immunosuppressive therapy can extend life expectancy, but it is not sufficient to prevent disease progression or associated complications.

#### CD 25 Deficiency

CD25 deficiency or interleukin two receptor alpha deficiency is caused by mutations in the *interleukin 2 receptor alpha* (*CD25) (IL2RA*) gene. The mutations result in expression of a defective α chain or a lack of CD25 causing variable extent of autoimmunity (9). Disease symptoms are overlapping with those of IPEX. To our knowledge there are no published data on alloHSCT in CD25 Deficiency.

#### LRBA Deficiency

Mutations in *LRBA* have been reported as causative for a rather complicated multi-organ autoimmunity syndrome with a highly variable clinical phenotype (1, 3, 10–12). LRBA protein is involved in protein endocytosis and vesicle transportation (10), the latter being a requirement for the surface expression of CTLA-4 (13), another immunoregulatory protein. Consequently, the symptoms of LRBA and CTLA-4 deficiency syndromes are largely overlapping. Affected individuals typically present with severe enteropathy in early life as (very) early onset inflammatory bowel disease (V)EOIBD, and may suffer from cytopenias, hypogammaglobulinemia, pulmonary disease, lymphoproliferative disorder, and infancy-onset type 1 diabetes mellitus. The risk of malignancies such as lymphoma is increased (3, 14). The same mutations in related or unrelated patient kindreds can present with entirely different symptoms and severity, thus, the clinical course of each patient remains rather unpredictable (1). Abatacept (see below) and sirolimus were shown to be effective for most symptoms in many individuals reported, who, however, remained dependent on this treatment (1, 10). Nonetheless, severely affected individuals have a significantly reduced quality of life and show early mortality by the disease or the complications of long-term immunosuppression. Therefore, alloHSCT as a potentially curative treatment option for these patients should be considered early.

Several case studies on successful alloHSCT in LRBA deficiency are available, reporting the use of reduced toxicity conditioning and serotherapy with a continuously rising number of at least 25 single or small patient series reports (15–18).

An ongoing international multicenter study, comparing the disease burden and long-term outcomes of conventional treatment with that of alloHSCT, includes data from 24 transplanted and >50 untransplanted patients (*V. Tesch and M.G. Seidel for the ESID Registry and the EBMT Inborn Errors Working Parties, unpublished data*), still showing a high transplant-related mortality of ~30%. Reasons of deaths were not specific for LRBA deficiency but included infections, thrombotic microangioapthy, toxicity and other reasons similar to a previous retrospective study (17). This might be in part be due to the fact that “historical” patients in poor pre-HSCT condition, without availability of targeted treatment and a genetic diagnosis at time of alloHSCT, were still included in this cohort. Both busulfan/melphalan and treosulfan with or without thiotepa have been used, and fludarabine was included as immunosuppressive backbone in almost all conditioning regimens reported. A healthy heterozygous donor can be used as a donor, although some heterozygous individuals were found to have autoantibodies against a variety of tissues, and residual autoimmunity in some patients persisted even after successful allogeneic HSCT (18). To date, however, published datasets and cohorts are too heterogenous and not large enough to allow any recommendation regarding the exact composition of chemotherapy drugs and the necessary level of donor chimerism for curing LRBA deficiency.

#### CTLA-4 Deficiency (Haploinsufficiency)

Pathogenic mutations in *CTLA-4* result in CTLA-4 haploinsufficiency causing an autosomal-dominant complex immune dysregulation syndrome with an incomplete penetrance. Clinical symptoms are largely overlapping with those of IPEX and LRBA deficiency with a variable symptom complex including early onset enteropathy, lymphoproliferation, organ infiltration, autoimmune cytopenias, and B-lymphocyte abnormalities, hypogammaglobulinemia, granulomatous lymphocytic interstitial lung disease, autoimmune thyroiditis, and arthritis (19, 20). Recently cancer prevalence of 12.9% was documented among 184 CTLA-4 deficient patients, highlighting the increased risk for malignant transformation, especially EBV^+^ lymphomas and gastric cancers (4). Abatacept, a CTLA-4–immunoglobulin fusion protein, ameliorates disease symptoms in CTLA-4 (and LRBA deficiency) to variable extent, as abatacept is a soluble version of CTLA-4 itself and is considered as a CTLA-4 replacement therapy (21). A few patients were reported receiving abatacept for gastrointestinal disease with partial improvement with a dosing of 30 mg/kg 2-weekly (bi-weekly). Belatacept might be an alternative. A recent publication on 22 LRBA-deficient patients receiving abatacept (children and young adults) showed that 16 patients achieved long-term complete control of lymphoproliferation and chronic diarrhea, especially autoimmune cytopenias. Weekly or bi-weekly administration of abatacept were associated to a better disease control than 4-weekly injections. There were no serious side effects related to the abatacept therapy. Circulating T follicular helper cell frequencies were found to be a reliable biomarker of disease activity, which decreased on abatacept therapy in most subjects (22). Studies on belatacept treatment, especially for pediatric patients are not available yet. Further prospective studies are crucial in order to assess the clinical value of abatacept/belatacept treatment as monotherapy and/or a bridge to transplant for severely affected CTLA-4 patients.

Large studies on transplanted CTLA-4 patients are not available yet. In a cohort of eight patients, seven individuals could be rescued by alloHSCT, with six patients having a complete remission of their symtoms and one patient showing partial response. RIC included combinations of treosulfan plus fludarabine with or without thiotepa, but also fludarabine plus melphalan and serotherapy. In this cohort, four of eight patients experienced GVHD despite having well-matched donors and receiving appropriate GVHD prophylaxis in three out of four cases. Based on their personal experience, the authors in this study suggested that a high level of inflammation in these patients might have contributed to the development of alloreactivity and so future patients are likely to benefit from either enhanced pre-HSCT immunosuppression or more aggressive post-HSCT GVHD prophylaxis (23).

Schwab et al. reported five transplanted patients among 90 affected CTLA4 mutation carriers undergoing alloHSCT between 10 and 50 years of age, due to uncontrollable cytopenia, enteropathy, and lymphoma with additional autoimmune disorders involving lymphoproliferative and infectious complications (20).

#### STAT3 GOF

Somatic activating gain-of-function (GOF) STAT3 mutations in the SH2 domain have been described in patients with T-lymphocyte and NK- cell Large Granular Cell Leukemia characterized by adult-onset lymphoproliferation, as well as autoimmunity with immune-mediated cytopenias (24). Genome wide association studies (GWAS) have also linked a STAT3 polymorphism to inflammatory bowel diseases (IBD) (25). In this manuscript, we focus on heterozygous germline activating mutations in *STAT 3* (STAT3 GOF) as a cause of a syndrome of early onset autoimmunity and lymphoproliferation with highly variable penetrance. Symptoms include early onset enteropathy, lymphocytic interstitial lung disease, and autoimmune cytopenias, associated with growth delay in some patients, endocrinopathies (diabetes), hepatic dysfunction, and susceptibility to opportunistic infections including mycobacterial disease and retention of deciduous teeth. On laboratory work up, hypogammaglobulinemia, associated with decreased switched memory B lymphocytes, NK cells and plasmacytoid dendritic cells have been reported as well as T-lymphocytopenia, dendritic cell deficiency, variable Th17 lymphocyte numbers, and low circulating eosinophils were observed as well (26–28). Despite the connection between increased STAT3 activity and neoplastic disease, as well as the observation that somatic GOF mutations in STAT3 lead to malignancies, neoplastic disease was quite rare in previously reported cohorts. One patient developed T-lymphocyte large granular lymphocytic leukemia at age 14. With regard to treatment options, a blockade of IL-6 activation with tocilizumab seems to ameliorate disease symptoms intermittently by reduction of TH-17 numbers to normal levels. Recent data show that JAK-inhibitors (e.g., ruxolitinib) could be an interesting alternative with positive impact on disease symptoms (29, 30).

There are published data on alloHSCT in two patients; one was curative with complete resolution of autoimmune symptoms and the other patient died of adenovirus infection and refractory graft vs. host disease (28). We are aware of unpublished data from 18 STAT3 GOF patients from 11 centers (international survey) who received alloHSCT with 61% survival. Causes of death were mainly infection complications and GVHD. Most of the patients received combinations of fludarabine plus treosulfan or busulfan and serotherapy in a myeloablative reduced toxicity manner. There was no superior conditioning *(Personal communication with J. Heimall and L. Forbes Satter)*. Of note, growth retardation by STAT3 GOF in some of the affected patients might remain uncorrected by alloHSCT.

#### BACH 2 Deficiency (Haploinsufficiency)

BACH 2 is a transcription factor participating in oxidative stress-mediated apoptosis and is involved in macrophage-mediated innate immunity as well as the adaptive immune response. It is essential for T- and B-lymphocytes. Single nucleotide variants in the *BACH2* locus associate with multiple autoimmune diseases. BACH 2 haploinsufficiency was recently reported causing primary immunodeficiency syndrome of immunodeficiency and autoimmunity, especially with intestinal inflammation (31). To our knowledge larger cohorts of BACH2 deficient patients and/or experience with alloHSCT are not published yet.

### Autoimmunity Disorders (APECED, ITCH-, ZAP70-, TPP2-, JAK1 GOF, and Prolidase Deficiency)

This section describes PID with autoimmunity including monogenetic diseases of the immune system affecting self-tolerance by thymic impairment (e.g., APECED) and/or other T-lymphocyte function deficiencies (ZAP70), syndromic diseases (JAK1 GOF) or metabolic diseases (Prolidase deficiency) (Table 2).

**Table 2:** Autoimmunity with or without Lymphoproliferation.

**Disease (*gene*)**	**OMIM/Inheritance**	**Clinical features**	**Targeted treatment**	**Risk of malignancy**	**Evidence of HSCT (reported cases *n* < 10, 10–100, >100)**
APECED (*AIRE*)	607358 AR/AD	Polyendocrinopathy Candidiasis Ectodermal dystrophy	No	Carcinoid	Not reported, no indication
ITCH deficiency *(ITCH)*	606409 AR	Early onset chronic lung disease Thyreoiditis Type I diabetes Developmental delay Dysmorphic features	No	Not reported	Not reported
TPP2 *(TPP2)*	190470 AR	Lymphoproliferation Severe autoimmune cytopenias Hypogammaglobulinemia	No	Not reported	<10
Prolidase deficiency (*PEPD*)	6132330 AR	Skin ulcers of lower extremities Teleangiectasias Recurrent infections dysmorphic face Intellectual disability Hepatomegaly Splenomegaly Anemia, thrombocytopenie, hypergammaglobinemia	No	Not reported	Not reported
ZAP-70 (*ZAP70*)	176947 AR	Combined immunodeficiency Autoimmunity	No	Lymphomas	10–100
JAK 1 GOF *(JAK 1)*	147795 AD	Hypereosinophilia Hepatosplenomegaly Failureto thrive	JAK inhibitors	Not reported	Not reported

There is no indication for alloHSCT for APECED (AIRE deficiency), since there is an intrinsic impairment of thymic function. Management includes antifungal therapy and symptomatic treatment of associated endocrine and autoimmune abnormalities.

#### ITCH Deficiency

*ITCHY E3 Ubiquitin protein ligase (ITCH)* encodes for ITCH protein transferring ubiquitin from E2 ubiquitin-conjugating enzymes to protein substrates. ITCH targets specific proteins for lysosomal degradation and plays a role in multiple cellular processes including erythroid and lymphoid cell differentiation and the regulation of immune responses. Mutations in this gene are a cause of syndromic multisystem autoimmune disease. To our knowledge, the largest reported cohort includes 10 patients with an homozygous mutation in *ITCH* having autoimmunity and morphologic and developmental abnormalities (32). Characteristical clinical features included dysmorphic facies, macrocephaly, failure to thrive, hepatomegaly, splenomegaly, and delayed motor development. Six of ten patients required enteral nutrition via gastrostomy tube placement within the first year of life due to failure to thrive, although only two had severe symptoms of malabsorption. There are no reported cases of alloHSCT for ITCH deficiency yet.

#### TPP2 Deficiency

*Tripeptidyl Peptidase 2 (TPP2)* is coding the TPP2 protein which is a serine exopeptidase involved in extralysosomal peptide degradation. Its deficiency in mice activates cell death programs and premature senescence. Likewise, the human TPP2 deficiency is a PID linking premature immunosenescence to severe autoimmunity. The first description of this disease included two affected siblings. One developed severe viral infections (HSV, VZV) in addition to autoimmunity, leading to the indication for alloHSCT at 10 years of age. AlloHSCT was performed using fludarabine, targeted low levels of busulfan, thiotepa, and antithymocyte globulin. Disease symptoms could be cured by alloHSCT (33).

Larger series of transplanted TPP2 patients are not available yet. Of note, some TPP2 patients showed neurodevelopmental delay. A recent study of a large cohort of patients with multiple sclerosis (MS) could identify TPP2 mutations as causative in three affected siblings of a consanguineous Syrian family. Authors concluded that a sterile brain inflammation due to TPP2 deficiency mimicks MS (34). Whether these patients would benefit from alloHSCT has not been assessed yet.

#### Prolidase Deficiency

Prolidase deficiency is a rare metabolic condition characterized by mutations in the *PEPD* gene resulting in disturbed collagene formation. Clinical symptoms include skin lesions, recurrent infections, unusual facial features, and variable intellectual disability. Patients seem to develop a variable degree of immunedysregulation, presenting as systemic Lupus erythematodes (SLE) as it was described in 10 patients having a SLE-like disease (35). Furthermore, a case of VEOIBD in presence of TTP2 deficiency was described in a toddler (36). We are not aware of any data on possible correction of disease symptoms by alloHSCT.

#### ZAP70

ZAP70 (zeta associated of 70 kDa) is a tyrosine kinase expressed by thymocytes, T- and NK- cells. ZAP70 deficiency results in a clinical picture of (severe) combined immunodeficiency (S)CID with abnormal T-cell receptor signaling and abnormal maturation and function of thymocytes. Characteristically, there are low to absent CD8^+^ lymphocytes with normal numbers of CD3^+^ and CD4^+^ T-lymphocytes; with disturbed response to mitogens and antigens.

Cuvelier et al. reported in a single center retrospective study of eight patients with confirmed ZAP70 deficiency receiving alloHSCT with and without conditioning. At a median follow-up of 13.5 years, all patients were alive. Patients receiving myeloablative conditioning regimens with busulfan and cyclophosphamide or fludarabine achieved and maintained 100 % chimerism for T-, B-lymphocytes and myeloid cells. Patients who received unconditioned transplants showed stable mixed donor chimerism for T-lymphocytes but engraftment of myeloid cells did not occur or was at least low in all of the patients. One patient had received ATG as serotherapy. Six of eight patients developed acute GVHD II/III, and two patients were reported having limited chronic GVHD, whereas two patients developed extensive cGVHD (resolved eventually). Seven of eight patients achieved stable IgG levels without need for immunoglobulin replacement (37).

Brager et al. collected data on 19 patients with ZAP70 deficiency of whom 16 patients underwent alloHSCT in seven different countries. Prior to alloHSCT all patients presented with repeated fungal and microbial infections and one patient with lymphoma. Patients who received myeloablative conditioning showed complete immune reconstitution, while lack of conditioning failed or resulted in partial immune reconstitution. In conclusion, these data showed favorable long-term outcomes in patients with ZAP70 deficiency receiving alloHSCT after conditioning (38).

#### JAK 1 GOF

The JAK/STAT interplay plays an important role in cytokine an growth factor signaling and controls hematopoiesis and immune function with activating somatic mutations being relevant for hematological myeloid malignancies (39). Germline JAK1 gain-of-function mutations cause a PID syndrome with autosomal dominant immune dysregulation and hypereosinophilia with eosinophilic infiltration of the gastrointestinal tract, massive hepatosplenomegaly and severe atopic dermatitis that can be successfully treated with ruxolitinib, an oral JAK1/2 inhibitor (40). To our knowledge there is no report on alloHSCT for JAK1 GOF to date.

### Immune Dysregulation With Colitis (IL-10-, IL10Ra-, IL10Rb, and NFAT 5-Deficiency)

In this section we discuss diseases with a distinc phenotype of inflammatory bowel disease including the “IL-10 group” of IL-10 and IL-10 receptor alpha and beta deficiency as well as the haploinsufficincy of NFAT5 (Table 3).

**Table 3:** Autoimmune dysregulation with colitis.

**Disease**	**OMIM/Inheritance**	**Clinical Features**	**Targeted treatment**	**Risk of malignancy**	**Evidence of HSCT (reported cases n <10, 10-100, >100)**
IL-10 deficiency (IL10) IL-10Ra deficiency IL-10Rb deficiency	124092 AR 146933 AR 123889 AR	Severe inflammatory bowel disease (abzesses, fistulae, fissures, need for colectomy), Chronic folliculitis, Respiratory diseases Arthritis	No	B cell lymphomas	10–100
NFAT5 haploinsufficiency (NFAT5)	604708 AD	Autoimmune enteropathy Antibodies against gut enterocytes and globlet cells, Eczematous rash Fever	No	Not reported	Not reported

#### IL10 and IL-10R a/b Deficiency

IL-10 being secreted by monocytes, macrophages, T-, B-lymphocytes, dendritic, epithelial and mast cells, is a crucial anti-inflammatory cytokine largely contributing to the maintenance of immune homeostasis via metabolic reprogramming of macrophages (41). Mutations in IL-10 and IL-10 R have been described as causative for severe VEOIBD (42, 43). Human inherited IL-10 receptor deficiency was also associated with a high risk of non-EBV–related diffuse large B-cell lymphoma suggesting that IL-10 signaling may be involved in the immune control of germinal center B-cell lymphoma (44). Engelhardt et al. presented a series of patients with IL-10 or IL-10R mutations suffering from symptoms of VEOIBD resulting in a complicated treatment-refractory IBD with fistulae, abscesses and significant fissures resulting in marked weight loss and growth retardation. This work included three transplanted patients, all being successfully treated resulting in complete remission of disease symptoms after myleoablative conditioning using blusulfan plus fludarabin and ATG in one patient, and fludradabine plus melphalan, and alemtuzumab in two others. All three patients showed stable engraftment with full donor chimerism without severe organ toxicity or higher grade GVHD. Authors concluded that because of the colitis being refractory to the standard immunosuppressive therapy, alloHSCT should be considered early as a curative therapeutic option (45). Kocacık Uygun et al. described two patients with IL-10 R deficiency, suffering from severe malnutrition and colon fistulae, who lacked a MSD and received alloHSCT from a MUD after MAC with busulfan (16 mg/kg), fludarabine (180 mg/m^2^) and ATG-Fresenius (30 mg/kg), and GVHD prophylaxis using cyclosporine and methotrexate (10 mg/m^2^ days 1, 3, and 6). Both patients showed an uncomplicated course of transplantation with remission of disease symptoms after early and stable engraftment with full donor chimerism (46).

Kotlarz et al. described five patients (out of a cohort of 16 IL10/IL-10R deficient patients) undergoing alloHSCT using a “standard” transplant protocol including alemtuzumab (1 mg/kg), fludarabine (180 mg/m^2^), treosulfan (42 g/m^2^), and thiotepa (10 mg/kg) and strict gut decolonization with colistin, amphotericin B (oral), ciprofloxacin, metronidazole, vancomycin (oral), and fluconazole. Furthermore, total parenteral nutrition was administered during the peritransplantation period. GVHD prophylaxis included cyclosporine and mycophenolate mofetil. All five patients survived without severe complications of alloHSCT. One patient needed a second transplantation (same donor) because of graft rejection at day +86 (47).

Karaca et al. reported a successful course of transplant in a female patient receiving reduced toxicity conditioning with busulfan (4 mg/kg/day) and fludarabine (40 mg/m^2^/day), and rabbit ATG (Fresenius) 10 mg/kg/day (48).

In conclusion, the course of the disease of these 11 transplanted patients is suggesting that early alloHSCT seems to be the only curative option for patients with IL-10/IL-10 receptor deficiencies. Reduced intensity or toxicity conditioning plus serotherapy should be used.

#### NFAT5

NFAT5 is a transcription factor with a pivotal role in regulating immune system in response to physiological and pathologic osmotic stress, even in relation to dieteray sodium excess. An essential role has been assigned to NFAT5 in immunity and autoimmune diseases, especially due to its known relevance for macrophage activation and survival, Treg generation, and TH17 differentiation. As a primary immunodeficiency syndrome, NFAT5 haploinsufficiency was recently described primarily presenting with IBD and autoimmunity (49), however, further published data on affected patients and on alloHSCT are not available to date.

## Discussion

The list of monogeneic immune dysregulative PID is rapidly expanding with the increasing availability of next generation sequencing. As our ability to establish a genetic diagnosis is improved, the challenge of linking genotype to phenotype and, consequentially, to a specific standard treatment, arises, a challenge that is made difficult by the small numbers of patients and complicated underlying pathophysiology of these diseases. Therefore, standardized treatment protocols, prognostication and unique treatment decisions are not available. Serious multi-organ autoimmunity and infectious complications of long-term immunosuppressive treatment can be life threatening. Today, targeted treatment such as abatacept or JAK inhibition might ameliorate symptoms in a porportion of patients, however, alloHSCT remains the only curative treatment option for most of the severely affected patients. It is to be expected that patients having multi-organ autoimmunity in their first decade of life, progress to a more severe disease complex with early manifestation of irreversible organ damage due to autoimmunity and complications of long-term immunosuppressive treatment. Therefore, the indication to proceed to alloHSCT remains a clinical decision, which (regardless of the final decision) should be evaluated in specialized pediatric PID-HSCT centers.

For IPEX, CTLA-4, and LRBA deficiency there is encouraging evidence for alloHSCT, especially if carried out early and when little “organ morbidity” with a low disease activity (8) is present. Various attempts to score the extent of immunodeficiency and dysregulation activity with one of the aims being to facilitate decisions to transplant have been and are being undertaken, e.g., within the retrospective IPEX study (8), the prospective study on profound combined immunodeficiencies (PCID) (50), and ongoing studies on CTLA-4 and LRBA deficiency (*unpublished data*). Whereas a common theme was that patients with advanced stages of poorly controlled PID with immune dysregulation (infections or autoimmunity) had worse outcomes in alloHSCT, one may argue that untransplanted patients will have a life-long, accumulating disease- and treatment-related toxicity burden, and a similarly unpredictable individual course as in alloHSCT. Thus, in conclusion, a patient with IPEX, LRBA deficiency, and probably also CTLA4 deficiency should undergo alloHSCT evaluation and donor search upon diagnosis, and, ideally, an attempt to ameliorate disease severity by targeted immunosuppressive treatment should be undertaken before alloHSCT. But the weaker a genotype-phenotype correlation and the better an available targeted treatment is, the more difficult will it be to counsel patient families on the specific transplant indication and urgency.

ZAP70 deficient patients might remain undetected in TREC-based Newborn Screening and present at later age with infectious complications and/or malignancies. However, alloHSCT should be considered early. RIC should be used to provide stable trilineage engraftment and immune reconstitution.

For STAT1/STAT3 GOF there is cumulative evidence for JAK inhibition, which should be initiated to evaluate the clinical response but also to reduce disease activity prior to alloHSCT. For patients suffering from IL-10/IL-10 receptor deficiencies, early alloHSCT should be carried out using reduced toxicity myeloablative or submyeloablative conditioning and serotherapy as there has been growing evidence throughout recent years showing unresponsiveness of colitis to the standard treatment.

## Author Contributions

SB and JF designed the study, provided data from literature, and wrote the manuscript. MS and EG revised the manuscript.

### Conflict of Interest

The authors declare that the research was conducted in the absence of any commercial or financial relationships that could be construed as a potential conflict of interest. The handling editor AL declared a collaboration with one of the authors, SB.

## Abbreviations

AUC: Area Under the Curve
alloHSCT: Allogeneic Haematopoeitic Stem Cell Transplantaiton
BACH2: BTB Domain And CNC Homolog 2
CTLA 4: Cytotoxic T lymphocyte Antigen 4
IPEX: Immunodeficiency, Polyendocrinopathy, X-linked
JAK: Janus Kinase
LRBA: LPS-responsive Beige Like Anchor Deficiency
MAC: Myeloablative Conditioning
NFAT5: Nuclear Factor of Activated T cells 5
RIC: Reduced Intensity Conditioning
STAT: Signal Transducers and Activators of Transcription
VEO-IBD: Very Early Onset Inflammatory Bowel Disease
ZAP70: Zeta Associated of 70 kDa.
